# Age and Latent Cytomegalovirus Infection Do Not Affect the Magnitude of De Novo SARS‐CoV‐2‐Specific CD8^+^ T Cell Responses

**DOI:** 10.1002/eji.202451565

**Published:** 2025-03-12

**Authors:** Jet van den Dijssel, Veronique A. L. Konijn, Mariël C Duurland, Rivka de Jongh, Lianne Koets, Barbera Veldhuisen, Hilde Raaphorst, Annelies W. Turksma, Julian J. Freen‐van Heeren, Maurice Steenhuis, Theo Rispens, C Ellen van der Schoot, S. Marieke van Ham, Rene A. W. van Lier, Klaas P. J. M. van Gisbergen, Anja ten Brinke, Carolien E. van de Sandt

**Affiliations:** ^1^ Sanquin Research and Landsteiner Laboratory, Amsterdam UMC University of Amsterdam Amsterdam The Netherlands; ^2^ Amsterdam Institute for Immunology and Infectious Diseases Amsterdam The Netherlands; ^3^ National Screening Laboratory of Sanquin Research and Laboratory Services Amsterdam The Netherlands; ^4^ Department of Immunohematology Diagnostics Sanquin Diagnostic Services Amsterdam The Netherlands; ^5^ R&D Sanquin Diagnostic Services Amsterdam The Netherlands; ^6^ Amsterdam UMC location Vrije Universiteit Amsterdam Molecular Cell Biology and Immunology Amsterdam The Netherlands; ^7^ Swammerdam Institute for Life Sciences University of Amsterdam Amsterdam The Netherlands; ^8^ Division LAB University Medical Center Utrecht Utrecht The Netherlands; ^9^ Physiology and Cancer Programme, Champalimaud Research Champalimaud Foundation Lisboa Portugal; ^10^ Department of Microbiology and Immunology University of Melbourne at the Peter Doherty Institute for Infection and Immunity Melbourne Victoria Australia

**Keywords:** aging, antigen‐specific CD8^+^ T cells, CMV, COVID‐19, immunosenescence

## Abstract

Immunosenescence, age‐related immune dysregulation, reduces immunity upon vaccinations and infections. Cytomegalovirus (CMV) infection results in declining naïve (T_naïve_) and increasing terminally differentiated (T_emra_) T cell populations, further aggravating immune aging. Both immunosenescence and CMV have been speculated to hamper the formation of protective T‐cell immunity against novel or emerging pathogens. The SARS‐CoV‐2 pandemic presented a unique opportunity to examine the impact of age and/or CMV on the generation of *de novo* SARS‐CoV‐2‐specific CD8^+^ T cell responses in 40 younger (22–40 years) and 37 older (50–66 years) convalescent individuals. Heterotetramer combinatorial coding combined with phenotypic markers were used to study 35 SARS‐CoV‐2 epitope‐specific CD8^+^ T cell populations directly ex vivo. Neither age nor CMV affected SARS‐CoV‐2‐specific CD8^+^ T cell frequencies, despite reduced total CD8^+^ T_naïve_ cells in older CMV^‐^ and CMV^+^ individuals. Robust SARS‐CoV‐2‐specific central memory CD8^+^ T (T_cm_) responses were detected in younger and older adults regardless of CMV status. Our data demonstrate that immune aging and CMV status did not impact the SARS‐CoV‐2‐specific CD8^+^ T cell response. However, SARS‐CoV‐2‐specific CD8^+^ T cells of older CMV^‐^ individuals displayed the lowest stem cell memory (T_scm_), highest T_emra_ and PD1^+^ populations, suggesting that age, not CMV, may impact long‐term SARS‐CoV‐2 immunity.

## Graphical abstract

Immunosenescence and cytomegalovirus infection are suggested to impair protective immunity against novel pathogens. Despite reduced total CD8^+^ T_naïve_ cells in older CMV^‐^ and CMV^+^ individuals, our study found a broad and robust memory SARS‐CoV‐2‐specific CD8^+^ T cell response in both younger and older convalescent COVID‐19 donors regardless of CMV status.

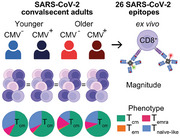

**Abbreviations**
A01: HLA‐A*01:01
A02: HLA‐A*02:01
A03: HLA‐A*03:01
A11: HLA‐A*11:01
A24: HLA‐A*24:02
B07: HLA‐B*07:02
B15: HLA‐B*15:01
B27: HLA‐B*27:05
B35: HLA‐B*35:01
B40: HLA‐B*40:01
CMV: cytomegalovirus
COVID‐19: coronavirus disease 2019
EBV: Epstein‐Barr virus
HTCC: heterotetramer combinatorial coding
pHLA‐I: peptides loaded onto the human leukocyte antigen class I molecule
SARS‐CoV‐2: severe acute respiratory syndrome coronavirus 2
StdDev: Standard Deviation
T_cm_
: central memory T cells
TCR: T cell receptor
T_em_
: effector memory T cells
T_emra_
: effector memory expressing CD45RA T cells
T_naive(‐like)_
: naïve (like) T cells
T_scm_
: stem cell memory T cells

## 1 Introduction

Age‐related dysregulated immunity, referred to as immunosenescence, is characterized by a reduction of B cells producing high‐affinity antibodies [1], declining naïve T cells [2, 3, 4], and accumulation of memory T cells, including senescent and exhausted T cells. The accumulation of memory T cells likely results from repeated pathogen exposure throughout human life [5]. Together, these age‐related changes reduce vaccine responsiveness, increase susceptibility to viral infections, and are associated with increased morbidity and mortality in older individuals [6]. The increase of the memory CD8^+^ T cell pool, a phenomenon known as memory inflation, is augmented in individuals latently infected with cytomegalovirus (CMV) through the accumulation of circulating CMV‐specific memory CD8^+^ T cells [7]. CMV seropositive individuals display higher CD8^+^ T cell frequencies and higher numbers of less functional terminally differentiated T cells (T_emra_) [2, 7–11]. On average, expanded CMV‐specific memory CD8^+^ T cells take up 10% of circulating memory T cells [11] and are thought to outcompete non‐CMV‐specific CD8^+^ T cells. This competition may hinder host defense mechanisms targeting other pathogens [12, 13, 14]. Overall, the effects of CMV on the immune system are complex, multifaceted, and pleiotropic [15]. The high global CMV prevalence (83%) [16] underscores the importance of understanding the influence of CMV on the formation and differentiation of CD8^+^ T cell responses directed against novel pathogens.

Antigen‐specific CD8^+^ T cells are essential in clearing viral infections and reducing disease severity [17, 18, 19]. The T cell receptor (TCR) of CD8^+^ T cells recognizes viral peptides (p) loaded onto human leukocyte antigen class I (HLA‐I) complexes present on the surface of infected cells. Epitope (pHLA‐I) recognition results in T cell activation, effector differentiation, elimination of virus‐infected cells, and memory formation. Antigen‐specific CD8^+^ T cells are initially selected from the naïve CD8^+^ T cell pool, which has a highly heterogeneous TCR repertoire that emerges during thymopoiesis [20, 21]. Thymic involution gradually hampers the replenishment of circulating naïve CD4^+^ and CD8^+^ T cells later in life, until it almost completely ceases after the age of 40 [20, 22, 23].

Older adults and CMV‐infected individuals demonstrate a decline in circulating naïve CD8^+^ T cells, potentially decreasing diversity within the naïve TCR repertoire, which may limit recognition of novel pathogens [2, 3, 21, 22, 24, 25]. Supporting this hypothesis, TCRs of Epstein–Barr virus (EBV)‐specific CD8^+^ T cells within CMV‐infected individuals were less diverse compared with CMV‐negative individuals [26]. Conversely, no differences were detected between TCR clonotype distribution of influenza‐specific CD8^+^ T cells in CMV‐seropositive (CMV^+^) and CMV‐seronegative (CMV^−^) individuals [27]. Furthermore, comparable EBV‐ and influenza‐specific CD8^+^ T cell frequency and function were described in human lymph nodes and peripheral blood. Conversely, CMV‐specific cells in the lymph nodes displayed a more polyfunctional memory phenotype compared with circulating cells [28], hinting at a tissue‐dependent effect of CMV. However, these studies did not address the impact of CMV or immunosenescence on de novo immune responses generated from naïve CD8^+^ T cells. By the age of 8 in Western societies, the seroprevalence of influenza (100% [29]) and EBV (54% [30]) is substantially higher compared with the seroprevalence of CMV (22% [31]). Therefore, the effect of aging and CMV status on primary CD8^+^ T cell responses directed against newly encountered pathogens in humans remains poorly defined. The SARS‐CoV‐2 pandemic provided a unique opportunity to separate the effect of age and CMV status on the generation of de novo antigen‐specific CD8^+^ T cell responses in humans.

Here we analyzed the impact of age and CMV on the generation of de novo SARS‐CoV‐2‐specific CD8^+^ T cell responses in 40 younger and 39 older convalescent adults following their first SARS‐CoV‐2 infection. In line with previous studies [2, 8, 9, 25, 32, 33, 34, 35, 36], we detected an age‐dependent decrease of total CD8^+^ T_naïve_ cells and a CMV‐driven increase in the total CD8^+^ T_emra_ population. However, robust frequencies of SARS‐CoV‐2‐specific CD8^+^ T cell populations expressing a clear central memory phenotype were induced after the initial SARS‐CoV‐2 infection, regardless of age or CMV status. This implies that reduced total naïve CD8^+^ T cell frequencies in younger individuals with latent CMV infection and older individuals independent of CMV status did not impede the formation of cellular immune responses against novel viruses such as SARS‐CoV‐2.

## 2 Results

### 2.1 Characteristics of the Convalescent Study Cohort

**FIGURE 1:**
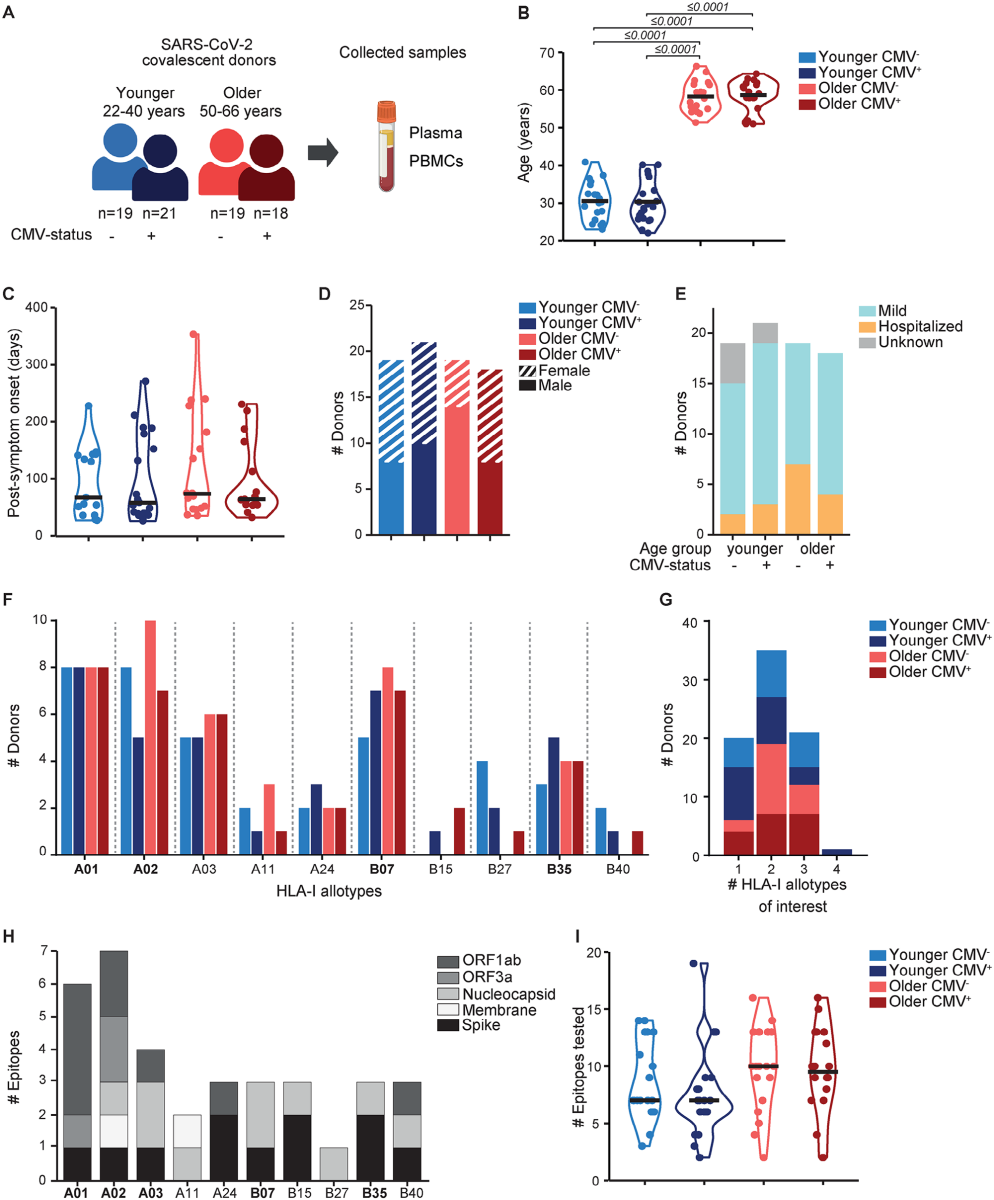
Convalescent COVID‐19 cohort and HLA‐I profiles. (A) Overview of convalescent donor cohort. Distribution of age (B), days post onset symptoms (C), sex (D), and disease severity (E). (C) Donors with unspecified days post‐onset symptoms were not included (younger CMV^−^ *n* = 4 and younger CMV^+^ *n* = 2 adults). (F) Number of donors with HLA‐I allele of interest per donor group, donors were selected based on the expression of at least one of the four prominent HLA‐I allotypes (bold). (G) Number co‐expressed HLA‐I allotypes of interest among donors in the cohort. (H) Protein distribution of 35 SARS‐CoV‐2 epitopes per HLA‐I allotype. (I) Number of SARS‐CoV‐2‐derived epitopes tested per donor group. (B, C, I) Each dot represents an individual donor (younger CMV^−^ *n* = 19, younger CMV^+^ *n* = 21, older CMV^−^ *n* = 19, and older CMV^+^ *n* = 18 adults). (B) Statistical analysis was performed using a one‐way ANOVA followed by the Tukey HSD test, horizontal bar indicates the mean. (C, I) Statistical analysis was performed using a Wilcoxon rank‐sum test with Bonferroni‐Holm's multiple comparison correction, horizontal bar indicates median. Significant *p‐*values are provided above the graph. (A) Created with BioRender.com.

To study the effect of age and latent CMV infection on the infection‐induced SARS‐CoV‐2 immune response, we determined the CMV‐seroconversion status of 40 younger (22–40 years) and 37 older (50–66 years) SARS‐CoV‐2 seroconverted convalescent adult donors (Figure 1A; Table S1). The 19 CMV^‐^ (mean 31.8 years, StdDev 5.6 years) and 21 CMV^+^ (mean 30.6 years, StdDev 5 years) younger adults were of similar age, likewise, no age difference was observed between 19 CMV^‐^ (mean 57.7, StdDev 3.3 years) and 18 CMV^+^ (mean 59.8 years, StdDev 2.9 years) older adult donors (Figure 1B). Convalescent donors were recruited in the spring of 2020 during the first wave of the SARS‐CoV‐2 pandemic. On average, blood samples were collected 102 days post‐symptom onset (range 26–353 days) with no significant differences between groups (younger CMV^‐^ median 67 days, younger CMV^+^ 58 days, older CMV^‐^ 74 days and older CMV^+^ 64 days; Figure 1B). Male participants were more prevalent in CMV^‐^ older adults (74%), but not in other donor groups (younger CMV^−^ 42%, younger CMV^+^ 48%, older CMV^+^ 44%; Figure 1D). Most donors recovered from mild disease (younger CMV^−^ 68.4%, younger CMV^+^ 76.2%, older CMV^−^ 63.2%, and older CMV^+^ 77.8%) while some were hospitalized (younger CMV^−^ 10.5%, younger CMV^+^ 14.3%, older CMV^−^ 36.8%, older CMV^+^ 22.2% CMV^+^). Six younger donors did not disclose their disease severity and were therefore labeled as unknown (younger CMV^−^ 21.1% and younger CMV^+^ 9.5% CMV^+^; Figure 1E).

In‐depth analysis of de novo SARS‐CoV‐2‐specific CD8^+^ T cell responses required well‐matched HLA‐I profiles between groups. Therefore, all donors were HLA‐I typed, revealing 10 prominent HLAs compatible with our assay, namely HLA‐A*01:01 (A01), HLA‐A*02:01 (A02), HLA‐A*03:01 (A03), HLA‐A*11:01 (A11), HLA‐A*24:02 (A24), HLA‐B*07:02 (B07), HLA‐B*15:01 (B15), HLA‐B*27:05 (B27), HLA‐B*35:01 (B35), and/or HLA‐B*40:01 (B40) (Figure 1F). We aimed to select at least six donors per group expressing A01, A02, B07, and/or B35, as these HLAs are highly prevalent within the Dutch population and/or were previously associated with dominant SARS‐CoV‐2‐specific CD8^+^ T cell immunity [37, 38, 39, 40, 41, 42, 43]. Due to lower allelic frequency, less than six B35 donors were included in all groups (3 younger CMV^‐^, 5 younger CMV^+^, 4 older CMV^−^, and 4 older CMV^+^ B35 expressing donors). Furthermore, only five B07^+^ CMV^−^ and A02^+^ CMV^+^ younger individuals could be selected (Figure 1F). Additionally, donors were preferably included when they simultaneously expressed multiple HLAs compatible with our assay, as a result, 79% of our donors co‐expressed multiple HLAs of interest, namely 2 (*n* = 34), 3 (*n* = 24), or 4 (*n* = 3) HLA‐I allotypes (Figure 1G).

In‐depth analysis was performed on CD8^+^ T cells specific for previously identified epitopes, including 35 SARS‐CoV‐2‐derived epitopes against which no pre‐existing immunity was reported [37, 39–52], 6 CMV, 2 Epstein‐Barr virus (EBV), and 2 influenza virus‐derived epitopes across the 10 HLA‐I allotypes (Table S2, Table S3). The 35 SARS‐CoV‐2 peptides were derived from spike (S; *n* = 11), ORF1ab (*n* = 9), ORF3a (*n* = 3), nucleocapsid (N; *n* = 10), and membrane protein (M; *n* = 2), with up to seven epitopes tested per HLA‐I allotype (average 4 epitopes per HLA‐I; Figure 1H). The number of SARS‐CoV‐2 epitopes tested was similar between all groups (median 7 epitopes in younger CMV^‐^ and CMV^+^ donors, 10 epitopes in older CMV^‐^ and CMV^+^ donors; Figure 1I).

The HLA‐I profiles of the convalescent donors provide a unique opportunity to study the de novo SARS‐CoV‐2‐specific CD8^+^ T cell responses across multiple epitopes in HLA‐matched CMV^+^ and CMV^−^ age groups.

### 2.2 Newly Induced SARS‐CoV‐2‐Specific CD8^+^ T Cell Frequencies Are Not Impacted by Age or CMV Status

**FIGURE 2:**
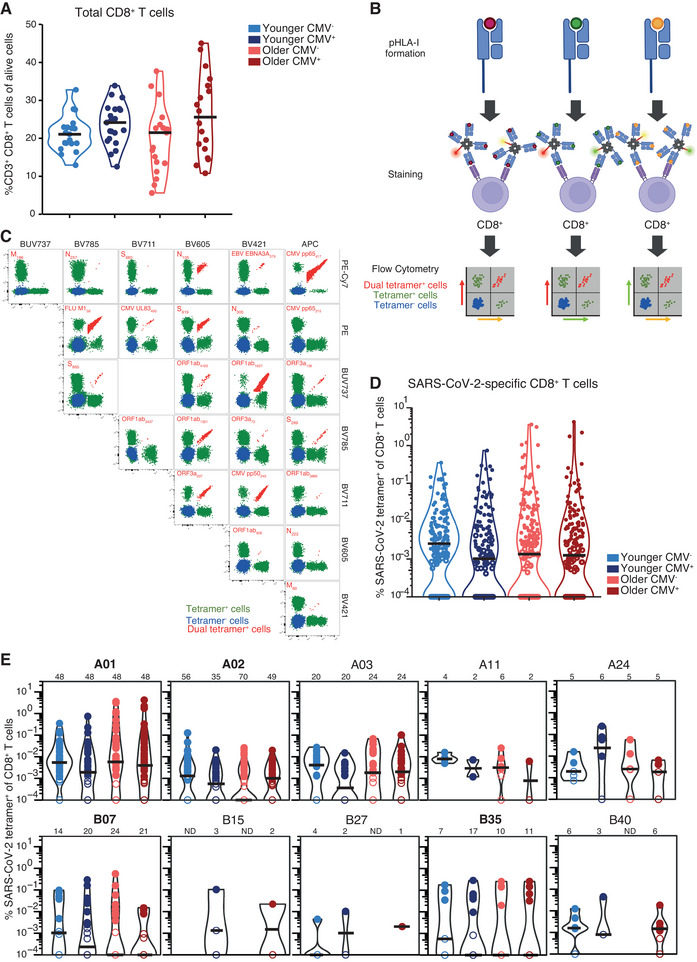
Robust SARS‐CoV‐2 CD8^+^ T cell frequencies independent of age and CMV status. (A) CD8^+^ T cell frequencies, each dot represents an individual donor. (B) Overview of the heterotetramer combinatorial coding approach, adjusted from [50]. (C) Representative flow cytometry plots created by Boolean gating CD8^+^ T cell populations expressing tetramer fluorophores (full gating strategy in Figure S2). Frequency of SARS‐CoV‐2 tetramer^+^CD8^+^ T cells per donor group (D) or subdivided per HLA‐I allotype (E), each dot represents an epitope‐specific population of an individual donor (younger CMV^−^ *n* = 19, younger CMV^+^ *n* = 21, older CMV^−^ *n* = 19 and older CMV^+^ *n* = 18 adults). (D, E) Open symbols indicate tetramer^+^CD8^+^ T cell populations of 3–8 detected cells, these were excluded from phenotypic analysis. Frequency of tetramer^+^CD8^+^ T cells is shifted by 10^−4^ to allow for visibility on logarithmic y axes (i.e., no detected tetramer^+^ events displayed as 10^−4^). (E) The number of HLA‐I‐restricted SARS‐CoV‐2 tetramer^+^CD8^+^ T cell populations are indicated at the top of the graph, not determined (ND) indicates HLAs for which no donors were available. The four prominent HLA‐I allotypes donors were selected for are indicated in bold. (A) Statistical analysis was performed using a one‐way ANOVA followed by the Tukey HSD test, horizontal lines indicate mean. (D, E). Statistical analysis was performed using a Wilcoxon rank‐sum test including Bonferroni‐Holm's multiple comparison correction, horizontal lines indicate median. No significant differences were identified. (B) Created with BioRender.com.

To test whether age and/or CMV impact the magnitude of the de novo SARS‐CoV‐2‐specific CD8^+^ T cell response in convalescent donors, we assessed the frequency of tetramer‐positive cells in the total CD8^+^ T cell population of each donor directly ex vivo. As the total CD8^+^ T cell population may affect how we respond to novel pathogens, we first assessed the frequency of the total CD8^+^ T cells in our cohort. No differences in total CD8^+^ T cell frequency were observed between age groups (Figure S1 and S2A). However, a significantly higher frequency of total CD8^+^ T cells was observed in CMV^+^ individuals compared with CMV^−^ individuals (Figure S2B), but this was not maintained when stratifying by age (Figure 2A).

Heterotetrametric Combinatorial Coding of pHLA‐I complexes was performed to probe the magnitude of multiple epitope‐specific CD8^+^ T cell populations simultaneously directly ex vivo, as per our recent studies [50, 53]. Epitope‐specific CD8^+^ T cells were defined as double‐positive for both fluorophores linked to a specific pHLA complex (Figure 2B,C; Figure S1). The technique allowed for the assessment of up to 26 epitopes simultaneously within a single individual, including as many as 19 SARS‐CoV‐2‐specific epitopes. The magnitude of the combined SARS‐CoV‐2‐specific CD8^+^ T cell population was similar between young and older CMV^−^ and CMV^+^ donors (Figure S2C,D). In addition, no differences were identified between the donor groups when stratifying by age and CMV status (Figure 2D; Figure S2E). Furthermore, the magnitude of the combined SARS‐CoV‐2‐specific CD8^+^ T cell populations remained stable >200 days post‐symptom onset, regardless of age or CMV status (Figure S2F), and no significant differences were identified across donors of different disease severity (Figure S2G). Together, these results indicate that a robust SARS‐CoV‐2‐specific CD8^+^ T cell response can be induced regardless of age or CMV status, which can be maintained over time. As anticipated, CMV‐specific CD8^+^ T cell frequencies were significantly higher in CMV^+^ individuals compared with CMV^−^ individuals but were similar between younger and older CMV^+^ adults (Figure S2H). No significant differences were observed for EBV‐ and influenza‐specific CD8^+^ T cells in the four donor groups (Figure S2I,J).

Individual SARS‐CoV‐2 epitopes have been shown to cover a range of immunodominance profiles and magnitudes [37–40, 53–55]. To correct for potential HLA‐I profile‐dependent differences between donor groups, SARS‐CoV‐2 epitope‐specific responses were assessed per HLA‐I allotype (Figure 2E) and revealed no differences between donor groups. Additionally, no significant differences could be identified across individual SARS‐CoV‐2 epitopes (Figure S3).

Together, these data demonstrated that neither age nor CMV status affected the induced magnitude of SARS‐CoV‐2 epitope‐specific CD8^+^ T cell responses.

### 2.3 Age and Latent CMV Infection Do Not Impact the Generation of SARS‐CoV‐2‐Specific T_cm_ CD8^+^ T Cells

**FIGURE 3:**
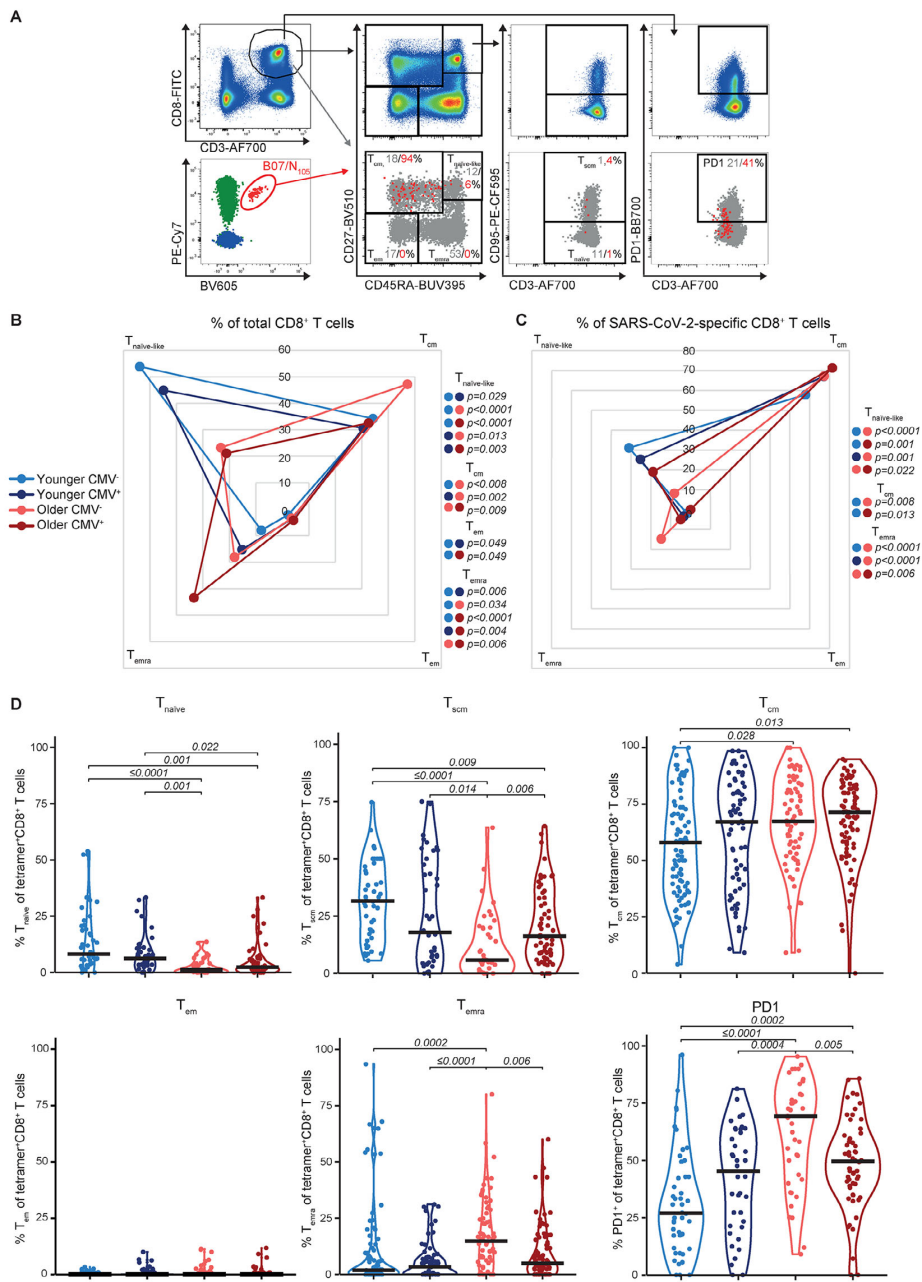
SARS‐CoV‐2‐specific CD8^+^ T cells display a robust T_cm_ phenotype regardless of age or CMV status. (A) Representative FACS gating strategy used to characterize the phenotype profile of SARS‐CoV‐2 epitope‐specific CD8^+^ T cells. CD27, CD45RA, CD95, and PD1 were used to identify T_cm_ (CD27^+^CD45RA^−^), T_em_ (CD27^−^CD45RA^−^), T_emra_ (CD27^dim/‐^CD45RA^+^), and T_naïve‐like_ (CD27^high^CD45RA^+^) cells, the latter can be further subdivided based on CD95 expression into T_naïve_ (CD27^high^CD45RA^+^CD95^−^) and T_scm_ (CD27^high^CD45RA^+^CD95^+^). Gates were defined based on the total CD8^+^ T cell population (top panel). The bottom panel displays overlays of the dual‐tetramer positive cells (red dots) on the entire CD8^+^ T cell population (grey dots). (B) Radar plots of median phenotype frequencies of the total CD8^+^ T cell per donor group (younger CMV^−^ *n* = 19, younger CMV^+^ *n* = 21, older CMV^−^ *n* = 19, and older CMV^+^ *n* = 18). (C) Radar plots of median tetramer^+^CD8^+^ T cell populations per donor group (younger CMV^−^ *n* = 19, younger CMV^+^ *n* = 18, older CMV^−^ *n* = 17, and older CMV^+^ *n* = 18). (D) Frequency of total T_naïve_, T_scm_, T_cm_, T_em_, T_emra_, and PD1^+^ tetramer^+^CD8^+^ T cell population per donor group, the horizontal bar indicates median (T_naïve_, T_scm_ and PD1 populations: younger CMV^−^ *n* = 8, younger CMV^+^ *n* = 10, older CMV^−^ *n* = 9 and older CMV^+^ *n* = 9 adults; T_cm_, T_em_, and T_emra_ similar numbers as in B). Statistical analysis was performed using a Wilcoxon rank‐sum test including Bonferroni‐Holm's multiple comparison correction. Significant *p*‐values are displayed next to the radar plots (B, C) or above the graphs (B–D).

The phenotype of total CD8^+^ T cells and SARS‐CoV‐2‐specific CD8^+^ T cells was assessed by analysis of phenotypic markers (CD27, CD45RA, and CD95; Figure 3A) to identify naïve‐like (T_naïve‐like_; CD27^high^CD45RA^+^), central memory (T_cm_; CD27^+^CD45RA^−^), effector memory (T_em_; CD27^−^CD45RA^−^) and CD45RA expressing terminally differentiated effector memory (T_emra_; CD27^dim/‐^CD45RA^+^) CD8^+^ T cells. In 38 out of 77 donors, additional staining for CD95 enabled us to further separate the CD8^+^ T_naïve‐like_ cell population into naïve (T_naïve_; CD27^high^CD45RA^+^CD95^−^) and stem cell memory (T_scm_; CD27^high^CD45RA^+^CD95^+^).

In line with previous findings [2, 3, 8, 9, 25, 32–36], age was the predominant factor associated with a reduction in total CD8^+^ T_naïve(‐like)_ cells (Figure 3B). This decline resulted from a decrease in the total CD8^+^ T_naïve_ cell population rather than the T_scm_ population (Figure S4A). In line with previous studies [2, 7, 11–15], total CD8^+^ T_emra_ cells were significantly higher in CMV^+^ individuals across both age groups and peaked in older CMV^+^ individuals (Figure 3B; Figure S4A). The total CD8^+^ T_cm_ frequency was significantly higher in older CMV^−^ individuals and may be attributed to repeated pathogenic infections/exposures across their lifetime (Figure 3B; Figure S4A). Meanwhile, the T_cm_ frequencies of CMV^+^ older individuals were comparable to younger donors, which may indicate that the T_cm_ phenotype is better preserved in CMV^−^ older adults (Figure 3B). Given the donors were healthy at the time of sampling, all donor groups expressed low frequencies of CD8^+^ T_em_ cells (Figure 3B; Figure S4A).

SARS‐CoV‐2‐specific CD8^+^ T cells had a predominant T_cm_ phenotype (younger CMV^−^ median 57.9%, younger CMV^+^ 67.1%, older CMV^−^ 67.3%, and older CMV^+^ 71.4%), regardless of age or CMV status, although significantly lower SARS‐CoV‐2‐specific CD8^+^ T_cm_ cells were observed in younger CMV^‐^ donors compared with older donors (CMV^−^ and CMV^+^; Figure 3C,D). The SARS‐CoV‐2‐specific CD8^+^ T_emra_ cell frequency was, compared with the total CD8^+^ T cells, relatively low, yet significantly higher in the CMV^‐^ older group (median 14.8%) compared with all other groups (younger CMV^‐^ median 1.9%, younger CMV^+^ 3.5%, and older CMV^+^ 5%; Figure 3C,D). In line with the total CD8^+^ T cell population, the frequency of SARS‐CoV‐2‐specific CD8^+^ T_naïve‐like_ cells was lower in older individuals compared with younger donors, with the lowest frequencies observed in older CMV^−^ individuals (Figure 3C). The decline could be attributed to a reduction of T_naïve_ and T_scm_ SARS‐CoV‐2‐specific CD8^+^ T cells, with a more pronounced decline in T_scm_ cells in older CMV^‐^ individuals (Figure 3D). Indeed, the SARS‐CoV‐2‐specific T_scm_ frequency negatively correlated with age in CMV^−^ individuals only (Figure 3D; Figure S4B). In contrast, PD1^+^ SARS‐CoV‐2‐specific CD8^+^ T cells positively correlated with age (Figure 3D; Figure S4C) and negatively correlated with the frequency of T_scm_ cells in both CMV^‐^ and CMV^+^ individuals (Figure S4D), which was predominantly observed in younger CMV^+^ and older CMV^‐^ individuals (Figure S4E). The SARS‐CoV‐2‐specific CD8^+^ T_naive‐like_ population was smaller in donors who were previously hospitalized, possibly as a result of a more robust T_cm_ and/or T_emra_ response in these donors. This trend was observed regardless of age or CMV status (Figure S4F).

Together these data demonstrated that the reduced total CD8^+^ T_naïve(‐like)_ population in older individuals did not impact the differentiation of de novo SARS‐CoV‐2‐specific CD8^+^ T cells into the long‐lived T_cm_ phenotype. However, the age‐related decrease in SARS‐CoV‐2‐specific CD8^+^ T_naïve_ and T_scm_ cells and increase of T_emra_ and PD1^+^ populations in older CMV^‐^ individuals, but less so in older CMV^+^ individuals, may indicate that memory may be affected over time.

## 3 Discussion

Antigen‐specific CD8^+^ T cells play a crucial role in viral clearance and reduction of disease severity [21, 22, 23]. However, individuals with a senescent immune system, as a result of aging and/or CMV‐induced memory inflation, are suggested to experience an impaired immune response against newly emerging viral infection or vaccination [25, 34]. Nevertheless, studying these effects in humans remains challenging. In this study, we demonstrate that the magnitude of the de novo SARS‐CoV‐2‐specific CD8^+^ T_cm_ response was not impacted by age or CMV status. Our data demonstrate that the effect of immunosenescence and CMV on the induction of CD8^+^ T cell immunity against newly emerging pathogens may be limited.

Aging is commonly linked to a reduction of the total CD8^+^ T cell population [2, 8], whereas multiple studies have shown an increase in CD8^+^ T cells in CMV^+^ individuals [8, 28, 35]. Here we find significantly higher frequencies of total CD8^+^ T cells in CMV^+^ individuals, but not in older individuals, potentially explaining the inability to detect significant differences among total CD8^+^ T cells when stratifying by both age and CMV status. This is in line with previous findings, which suggested that the increase in the total CD8^+^ T cell frequency in CMV^+^ individuals could be attributed to an expansion of the CMV‐specific CD8^+^ T cell population [2, 7, 9, 11].

In line with the literature, age was the predominant factor associated with a reduction of CD8^+^ T_naïve(‐like)_ cells in older individuals, which was not further impacted by their CMV status [2, 8, 9, 25, 32–36, 56]. Memory inflation of CD8^+^ T cells has been hypothesized to limit the immunological space against novel viruses in older and CMV^+^ individuals [11, 12, 13, 14]. However, we showed that the reduction in total CD8^+^ T_naïve_ cells did not affect the frequency of SARS‐CoV‐2 epitope‐specific CD8^+^ T cells in older and/or CMV^+^ individuals. These differences might be explained by a more pronounced CMV‐related memory inflation in circulation but less in tissue [28], allowing unhindered recruitment of novel SARS‐CoV‐2‐specific cells from lymph nodes during infection. Whether CMV infection impacts the composition of the epitope‐specific TCR repertoire in humans remains unclear. To verify whether CMV affects the T cell population at a clonal level, epitope‐specific paired TCRαβ sequencing would be required. However, a higher clonal diversity was found in CMV^+^ mice after OVA‐expressing *Listeria monocytogenes* [57].

We previously found that CMV does not affect the memory profile of influenza‐specific CD8^+^ T cells [21] in older and younger individuals. However, these individuals likely established influenza‐specific memory CD8^+^ T cells before their CMV infection and naïve T cell depletion. Indeed, we observed no differences in EBV or influenza‐specific CD8^+^ T cell frequencies across the different groups. The coronavirus disease 2019 (COVID‐19) pandemic provided a unique opportunity to access how a latent CMV infection affects CD8^+^ T cell memory formation directed against a novel viral infection. We examined the responses of SARS‐CoV‐2‐specific CD8^+^ T cells in individuals infected in the early stages of the COVID‐19 pandemic, ensuring analysis of a de novo immune response. Despite a reduced frequency of T_naïve_ cells in the total CD8^+^ T cell population, robust SARS‐CoV‐2‐specific CD8^+^ T_cm_ responses developed in individuals independent of age or CMV status. The formation of a robust T_cm_ memory population suggests that aged and/or CMV‐infected individuals can establish T cell‐driven protection against severe COVID‐19 upon subsequent SARS‐CoV‐2 infections [18, 58]. Studies in older mice indeed demonstrated that herpesvirus infections, including mouse CMV, did not impair immune responses and protection against acute emerging pathogens [59]. However, longitudinal studies are required to address the impact of CMV and age on the longevity and clinical benefit of CD8^+^ T cell responses against future SARS‐CoV‐2 infections and shaping the vaccine‐induced immune response over time.

In line with previous research, a decline in SARS‐CoV‐2‐specific CD8^+^ T_naïve_ cells in older convalescent individuals was observed [36, 49, 53]. Furthermore, to the best of our knowledge, we are the first to describe that SARS‐CoV‐2‐specific CD8^+^ T_scm_ cells were reduced in older CMV^−^ and CMV^+^ individuals, with the lowest frequencies of T_naïve_ and T_scm_ observed in older CMV^‐^ individuals. A similar trend, although not significant, was observed in young CMV^+^ individuals. In addition, SARS‐CoV‐2‐specific CD8^+^ T_emra_ and PD1^+^ populations were significantly increased in older CMV^‐^ individuals. Even though PD1 expressing SARS‐CoV‐2‐specific CD8^+^ T cells of convalescent donors were previously shown to be functional and not exhausted [60], the frequency of PD1^+^ SARS‐CoV‐2‐specific CD8^+^ T cells in our study was higher than described before. Interestingly, an inverse correlation of T_scm_ and PD1^+^ SARS‐CoV‐2‐specific CD8^+^ T cell populations was observed. Contrary to expectation, the proinflammatory environment previously described in latent CMV infection [61, 62, 63] may not guide non‐CMV‐specific CD8^+^ T cells toward a terminally differentiated phenotype. Instead, it potentially counteracts the age‐related dysregulation of the immune response, as older CMV^+^ individuals had lower frequencies of PD1^+^ and T_emra_ SARS‐CoV‐2‐specific CD8^+^ T cells compared with older CMV^‐^ individuals. Further analysis with comprehensive activation, exhaustion and functional analysis are needed to identify whether the observed terminally differentiated CD8^+^ T cells in CMV^−^ older individuals are truly exhausted and if they require special consideration in vaccination strategies.

Throughout life, repeated pathogen exposure results in an accumulation of memory CD8^+^ T cells. These cells may exhibit cross‐reactivity against novel pathogens, which could potentially compensate for the age‐dependent change in the de novo T cell population. Although cross‐reactivity with other pathogens has been described for certain SARS‐CoV‐2 epitope‐specific T cells [36, 64–66], it is unlikely that cross‐reactivity influences our findings, as we did not include any of the previously identified cross‐reactive epitopes in our analysis.

Reduced vaccine responsiveness is a common problem in older individuals [67], but the effect of CMV on vaccination responsiveness remains controversial [68] as reports showed reduced [34, 69–71], similar [34, 69–71] or enhanced [71, 72, 73] antibody responses post‐vaccination. The impact of CMV on vaccine‐induced SARS‐CoV‐2‐specific CD8^+^ T cells is less well studied and contradictory, with one study describing a negative impact [56] while others report no effect [33] of CMV on SARS‐CoV‐2‐specific CD8^+^ T cell frequencies. However, these studies did not examine SARS‐CoV‐2 CD8^+^ T cell memory formation, nor did they correct for age or HLA‐I profile. Our study showed that age and/or CMV‐associated immunosenescence did not impact the frequency and central memory formation of a de novo CD8^+^ T cell response against SARS‐CoV‐2.

Together our findings demonstrated that neither age nor CMV status affected the formation of a robust SARS‐CoV‐2 epitope‐specific CD8^+^ T cell response. The implications of our findings extend beyond SARS‐CoV‐2, as they encourage the development of vaccines that induce CD8^+^ T cells against current and emerging viruses.

### 3.1 Data Limitations and Perspectives

Most participants recovered from mild disease; the cohort therefore might not represent the full spectrum of immune responses seen after severe disease. Latent CMV can reactivate in individuals with weakened immune responses, such as hospitalized COVID‐19 patients, which may impact novel SARS‐CoV‐2 immunity [74, 75]. Our cohort consists of relatively young individuals (max 66 years), further research is required to assess the effects of age and CMV in people over 70. People with lower socioeconomic status and certain ethnic backgrounds are at higher risk of CMV infection, both of which may have also impacted their SARS‐CoV‐2 specific immune response [76, 77]. We were unable to correct for these potential co‐founding factors as social economic and ethnic information unavailable. By HLA‐matching donor groups we aimed to reduce any potential effect of differentially expressed HLA‐I allotypes across different ethnic groups. Furthermore, multiple studies identified CMV‐seroconversion as a risk factor for severe COVID‐19, particularly in younger individuals. It remains unclear whether CMV‐associated changes in the immune system or CMV reactivation due to poor health can cause aggravated disease after SARS‐CoV‐2 infection [78, 79, 80]. In our study, no such effect was observed. Although our HTCC assay covered a broad range of 35 SARS‐CoV‐2 epitopes, none of which were reported as cross‐reactive, our assay could not capture the full magnitude of the de novo CD8^+^ T cell response. We likely missed other de novo epitopes, particularly those restricted by less common HLAs, as they are not yet identified and could therefore not be included in our study. Ideally, we would establish the absolute number of SARS‐CoV‐2‐specific CD8^+^ T cells per mL blood, rather than frequencies relative to the total CD8^+^ T cell population, as CMV infections may impact the total number of CD8^+^ T cells in the blood [2, 5]. However, whole blood samples required for such analysis were not available. Additional phenotypic markers such as CCR7 could have provided further details about T cell phenotype subsets. Due to the low frequency of the SARS‐CoV‐2‐specific CD8^+^ T cells, we were unable to verify and compare the functional properties of SARS‐CoV‐2‐specific CD8^+^ T cells in our donors directly ex vivo. Lastly, this study focused on the blood compartment during a single memory timepoint of convalescent donors, thereby excluding a potential effect of age and CMV during acute infection, longitudinally, or in other tissues.

## 4 Methods

### 4.1 Study Participants and Sample Collection

Convalescent SARS‐CoV‐2 donors infected in the spring of 2020 were recruited as part of the COVID‐19 Convalescent Plasma program at Sanquin Blood Supply Foundation, Amsterdam, Netherlands. We analyzed 77 convalescent donors, 39 of whom were previously included in an epitope discovery study [53]. Participant demographics are described in Table S1. All donors were recruited before June 2020 when SARS‐CoV‐2 PCR tests were not readily available, so SARS‐CoV‐2 seroconversion was confirmed by serology, as described previously [50]. Symptom onset, date of recovery, and disease severity were determined by a questionnaire, categorizing severities as mild (minimal symptoms, at home), hospitalized (in ward or intensive care unit), or unknown (no information provided). CMV‐seroconversion was determined by Sanquin Diagnostiek B.V.

Participation was voluntary and nonremunerated, and all participants provided written informed consent as part of the routine blood collection procedure of the Sanquin Blood Supply Foundation (Blood bank). The study is in accordance with the declaration of Helsinki, Dutch regulations, and approved by the Ethics Advisory Council of Sanquin Blood Supply Foundation.

Blood collection occurred at least 2 weeks post‐self‐reported COVID‐19 recovery. Ficoll‐Paque separation was used to isolate peripheral blood mononuclear cells (PBMCs) from heparinized peripheral blood or buffy coats, granulocytes were collected for HLA‐I typing and plasma for serology.

### 4.2 HLA‐I‐Typing

Granulocyte genomic DNA was extracted using the QIAamp DNA mini kit (Qiagen). Subsequent HLA class I genotyping was conducted by the Department of Immunogenetics of Sanquin Diagnostiek B.V. or the Axiom genotyping platform according to the Axiom Propel XPRES 384HT Workflow (Applied Biosystems) as described previously [50].

We aimed to select at least six donors expressing at least one of four prominent HLA‐I allotypes (A01, A02, B07, and/or B35) and preferably a combination of multiple of the 10 HLA allotypes of interest (A01, A02, A03, A11, A24, B07, B15, B27, B35, and/or B40).

### 4.3 CD8^+^ T Cell Epitopes

35 SARS‐CoV‐2‐specific CD8^+^ T cell peptides, 8‐ to 11‐ amino acids in length, against which no pre‐existing immunity was observed in prepandemic samples, were selected based on previous studies (Table S2) and 6 CMV, 2 influenza, and 2 EBV peptides were included as controls (Table S3; JPT). Peptide binding to their respective HLA‐I allotype was previously confirmed [50, 53]. For the previously published convalescent donors only data from overlapping epitopes was included [53].

### 4.4 Generation of Combinatorial Encoded pHLA‐I Tetramers

HLA‐I complexes with UV‐cleavable peptides were generated in‐house by Sanquin Immunomonitoring Services of Sanquin Diagnostiek B.V. as described previously [50, 53, 81 ^,^ 82]. In brief, recombinant heavy chains of the HLA‐A*01:01, ‐A*02:01, ‐A*03:01, ‐A*11:01, ‐A*24:02, ‐B*07:02, ‐B*15:01, ‐B*27:05, ‐B*35:01, and ‐B*40:01 alleles and the B2M light chain were produced in Escherichia coli. pHLA‐I complexes were assembled by combining the heavy chain, light chain, and UV‐cleavable peptides [83], which was followed by purification via gel‐filtration High‐Performance Liquid Chromatography (HPLC). The biotinylated UV‐sensitive pHLA‐I complexes were stored at −80°C until use. UV‐sensitive pHLA‐I complexes were subjected to 366 nm UV light facilitating UV‐mediated exchanges to acquire pHLA‐I complexes [81]. Next, distinct fluorescent streptavidin‐conjugates (APC, PE, PE‐Cy7 (ThermoFisher), BV421, BV605, BV711, BUV661, BUV737, PE‐CF595 (BD bioscience (BD)), and BV785 (Biolegend) were linked to the biotinylated pHLA‐I complexes, resulting in tetramer formation. The UV exchange and combinatorial coding techniques are patent‐protected in Europe, the US, and other countries WO 2010/060439 and WO 2006/080837.

### 4.5 Flow Cytometry

PBMCs were thawed in RPMI 1640 (Life Technologies) supplemented with 10% FCS (Bondinco), 1% l‐glutamine (Sigma), 1% penicillin‐streptomycin (Sigma), and 1:1000 DNase (Worthington Biochemical Corporation, 10 mg/mL). After washing in MACS buffer (0.5% BSA and 2 mM EDTA in PBS), 4–10 million cells per sample were resuspended in FACS‐buffer (0.1% NaN3 0.5% BSA (Sigma) in PBS, 0.2 µm filtered (Whatman). Donors were grouped per HLA‐I allotypes. In the presence of Brilliant Staining Buffer Plus (BD, cat. 566385) HLA‐I tetramer pools were generated for each group. The previously studied group (*n* = 39) were co‐stained with CD8‐FITC (clone SK1, BD, cat. 345772), anti‐human CD3‐AF700 (clone UCHT1, BD, cat. 557943), anti‐human CD45RA‐BUV395 (clone HI1000, BD, cat. 740298), anti‐human CD27‐BV510 (clone O323, BD, cat. 751672), and Near‐IR‐Dye (Invitrogen, cat. L10119). In addition to this panel, the antibody mix for the new donors (*n* = 38) also included anti‐human CD95‐PE‐CF595 (clone DX2, BD, cat. 562395), anti‐human PD1‐BB700 (clone EH12.1, BD cat. 566460), anti‐human HLA‐DR‐BUV496 (clone L203, BD, cat. 752493), and anti‐human CD38‐BUV805 (clone HB7, BD, cat. 742074). Cells were washed twice and fixated with IntraStain (Agilent Daka) following the manufacturer's instructions and resuspended in FACS buffer for acquisition on the BD FACSymphony A5 with FACSDiva software (BDs) and analyzed using FlowJo (V10.8.1) (Treestar) [84]. The detection threshold was set at ≥3 double tetramer^+^CD8^+^ T cells, while a threshold of ≥9 tetramer^+^CD8^+^ T cells was used for further phenotypic characterization, consistent with our previous study [53].

### 4.6 Statistics

Sample sizes were not determined by a statistical method but by the availability of HLA‐typed donors. Donors were selected based on their HLA‐I allotypes. Measurements were taken from distinct donor samples: however, all donors were stained with multiple tetramers, thus frequency and phenotype plots of tetramer‐specific CD8^+^ T cells may contain multiple tetramer populations from a single donor sample. Due to the limited availability of human samples, assays could not be repeated. All statistical analyses were performed in R (v4.2.2). Data were analyzed for normal distribution using the one‐sided Shapiro–Wilk test (shapiro.test function). All groups in a graph were compared unless stated otherwise. Statistical significance for nonparametric data or groups with <10 data points were analyzed using the two‐sided unpaired Wilcoxon rank‐sum test (wilcox_test function), corrected for multiple comparisons using the Bonferroni‐Holm method. The one‐way analysis of variance (ANOVA), followed by the two‐sided Tukey's Honest Significant difference (HSD) test, or the two‐sided Student's *t*‐test was used to assess statistical differences of normally distributed data (aov, TukeyHSD, and t.test function). Lastly, the two‐sided Spearman's rank‐order correlation was computed to assess the relationship between multiple parameters. Differences were considered significant at a *p*‐value of <0.05.

## Author Contributions

Carolien E. van de Sandt led the study. Rene A. W. van Lier, Klaas P. J. M. van Gisbergen, Anja ten Brinke, and Carolien E. van de Sandt supervised the study. Jet van den Dijssel, Klaas P. J. M van Gisbergen, Rene A. W. van Lier, and Carolien E. van de Sandt conceptualized the study. Jet van den Dijssel, Barbera Veldhuisen, Annelies W. Turksma, Maurice Steenhuis, Julian J. Freen‐van Heeren, and Carolien E. van de Sandt designed the experiments. Jet van den Dijssel, Veronique A. L. Konijn, Mariël C. Duurland, Lianne Koets, Maurice Steenhuis, Hilde Raaphorst, and Julian J. Freen‐van Heeren performed the experiments. Jet van den Dijssel, Lianne Koets, Barbera Veldhuisen, Annelies W. Turksma, and Jet van den Dijssel analyzed the data. Jet van den Dijssel, Veronique A. L. Konijn, Mariël C. Duurland, Rivka de Jongh, Maurice Steenhuis, S. Marieke van Ham, Theo Rispens, and Anja ten Brinke managed the convalescent cohort. C. Ellen van der Schoot, S. Marieke van Ham, and Anja ten Brinke acquired the financial support. Jet van den Dijssel performed statistical analysis and visualized the work. Jet van den Dijssel, C. Ellen van der Schoot, S. Marieke van Ham, Rene A. W. van Lier, Klaas P. J. M. van Gisbergen, Anja ten Brinke, and Carolien E. van de Sandt provided intellectual input into study design and data interpretation. Jet van den Dijssel, Rene A. W. van Lier, Klaas P. J. M. van Gisbergen, and Carolien E. van de Sandt wrote the manuscript. All authors reviewed and approved the manuscript.

## Conflicts of Interest

The authors declare no conflicts of interest. The UV exchange and combinatorial coding techniques are patent‐protected in Europe, the US, and other countries WO 2010/060439 and WO 2006/080837. During this study, MS affiliation changed to Central Committee on Research Involving Human Subjects (CCMO), The Hague, the Netherlands, and KPJMG changed to Champalimaud Foundation in Lisbon, Portugal. Relocations did not affect their conflicts of interest.

## Supporting information



Supporting Information

Supporting Information

Supporting Information

Supporting Information

## Data Availability

Donor data are available in Table S1. This study did not generate new unique reagents. Source data are deposited in Mendeley [doi: 10.17632/hfcrnhtmc3.1]. Additional data are available from the corresponding author upon reasonable request. No custom code or mathematical algorithms were used in the preparation of the manuscript.
