# Supplementary material for: Age and Latent Cytomegalovirus Infection Do Not Affect the Magnitude of De Novo SARS‐CoV‐2‐Specific CD8+ T Cell Responses
Source: Eur J Immunol. 2025 Mar 12;55(3):e202451565. doi: 10.1002/eji.202451565 (PMC11898545; doi:10.1002/eji.202451565)
Supplement: Supplementary file 1 — Supporting Information [file EJI-55-e202451565-s002.pdf]

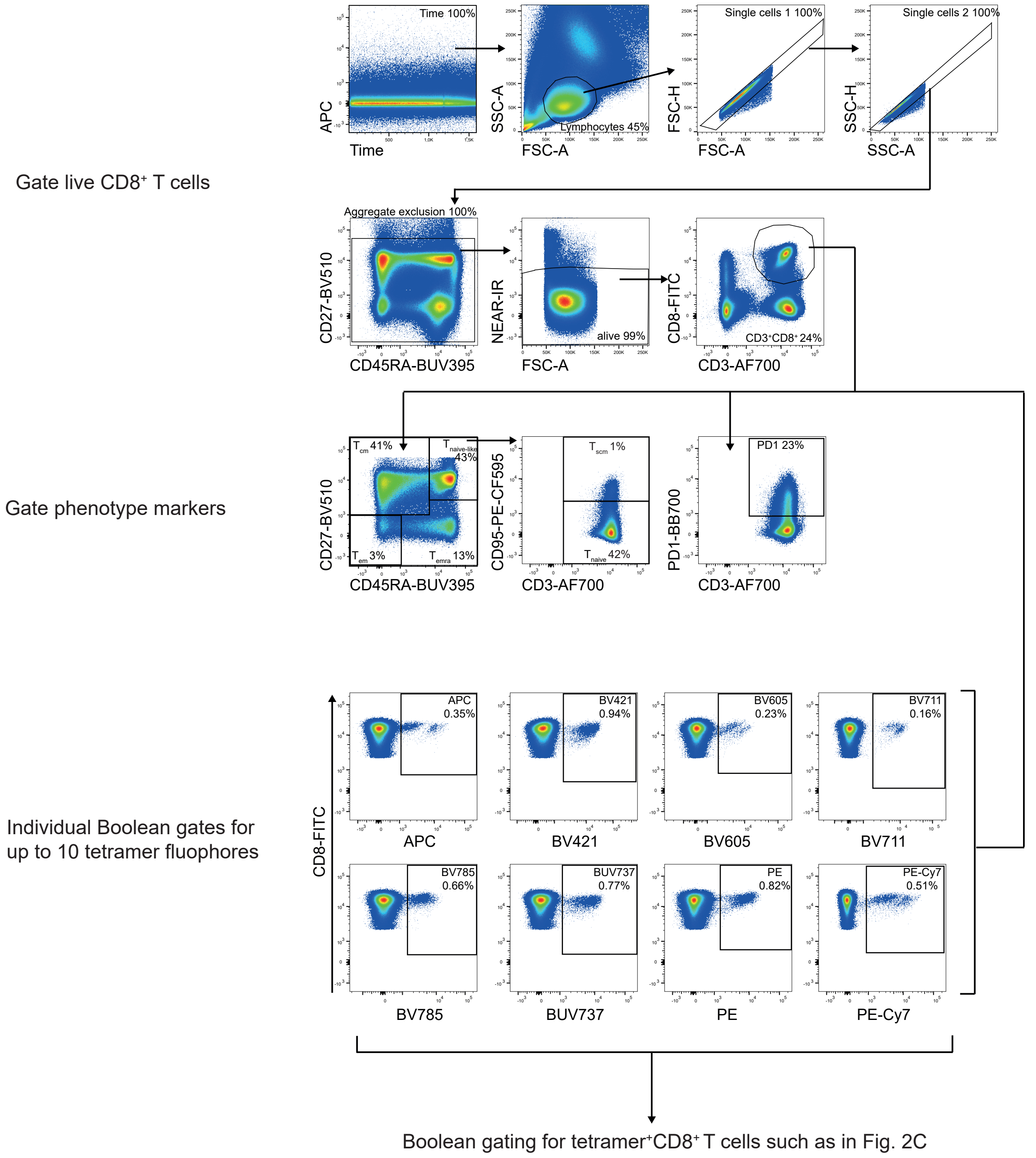

Figure S1. Gating strategy epitope-specific CD8$^+$ T cells
Representative gating strategy to identify SARS-CoV-2 epitope-specific CD8$^+$ T cells.

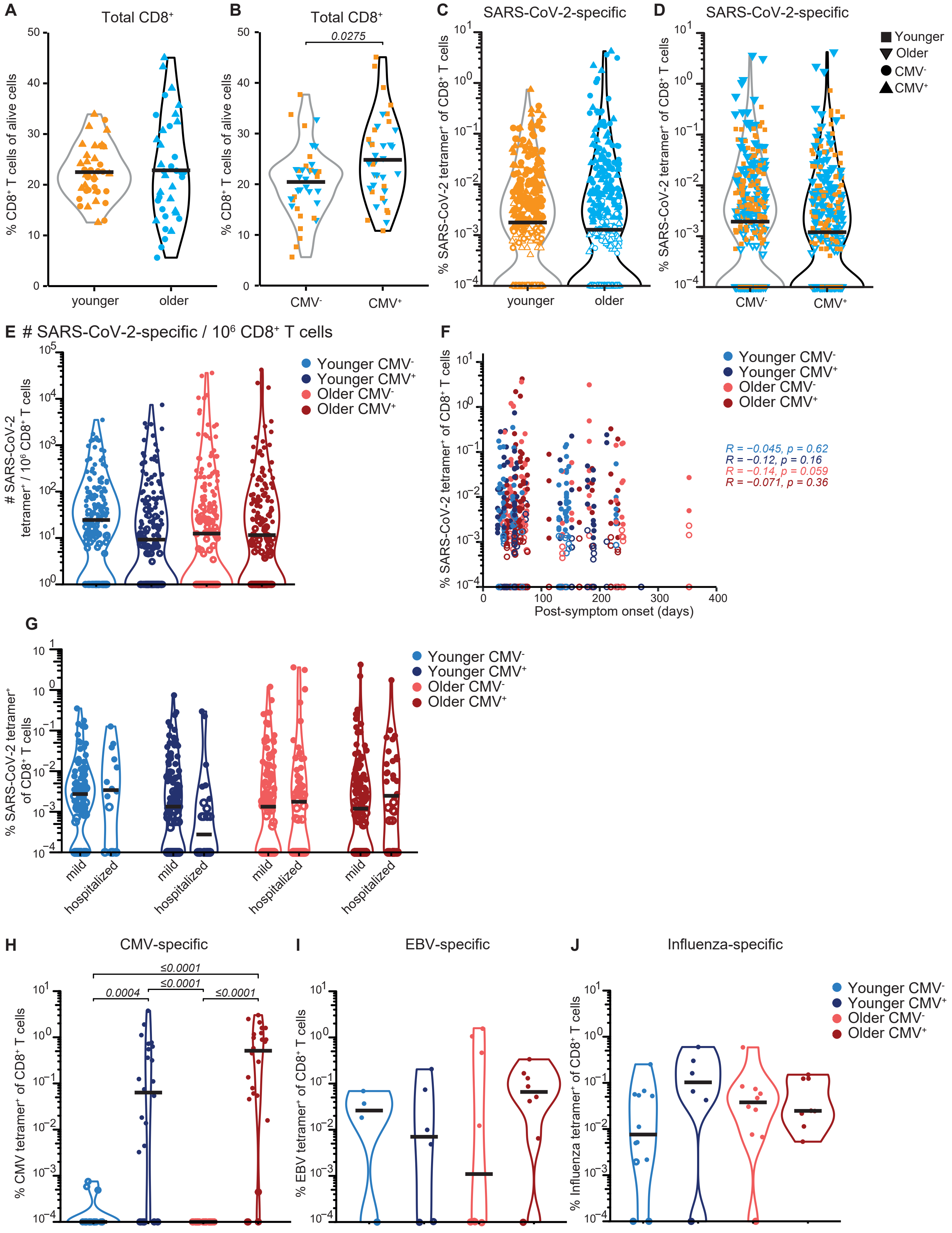
A
Total CD8+
% CD8+ T cells of alive cells
younger
older
B
Total CD8+
0.0275
CMV-
CMV+
C
SARS-CoV-2-specific
% SARS-CoV-2 tetramer+ of CD8+ T cells
D
SARS-CoV-2-specific
Younger
Older
CMV-
CMV+
E # SARS-CoV-2-specific / 10^6 CD8+ T cells
SARS-CoV-2 tetramer+ / 10^6 CD8+ T cells
Younger CMV-
Younger CMV+
Older CMV-
Older CMV+
F
R = −0.045, p = 0.62
R = −0.12, p = 0.16
R = −0.14, p = 0.059
R = −0.071, p = 0.36
Post-symptom onset (days)
G
% SARS-CoV-2 tetramer+ of CD8+ T cells
mild
hospitalized
H
CMV-specific
≤0.0001
0.0004
≤0.0001
≤0.0001
% CMV tetramer+ of CD8+ T cells
I
EBV-specific
% EBV tetramer+ of CD8+ T cells
J
Influenza-specific
% Influenza tetramer+ of CD8+ T cells

Figure S2. Total and tetramer$^+$CD8$^+$ T cell frequencies

CD8$^+$ T cell frequency by age group (A) or by CMV-status (B), each dot represents an individual donor. SARS-CoV-2 tetramer$^+$ cell frequency per age group (C) or CMV-status (D). (A-D) n=40 younger and n=37 older adults, n=38 CMV$^-$ and n=39 CMV$^+$ individuals. (E) Number of SARS-CoV-2 tetramer$^+$CD8$^+$ T cells per 10^6 CD8$^+$ T cells per donor group. Samples without tetramer$^+$CD8$^+$ T cells, were displayed as 1 for visibility on logarithmic y axes. (F) Spearman's rank-order correlation of the frequency of SARS-CoV-2-specific CD8$^+$ T cells with days post-symptom onset (PSO). (E,F) Younger CMV$^-$ n=19, younger CMV$^+$ n=21, older CMV$^-$ n=19 and older CMV$^+$ n=18 adults. (G) Frequency of SARS-CoV-2 tetramer$^+$CD8$^+$ T cells in mild and hospitalized patients per donor group (mild and hospitalized respectively: younger CMV$^-$ n=13 and n=2; younger CMV$^+$ n=16 and n=2; older CMV$^-$ n=12 and n=7; older CMV$^+$ n=14 and n=4). Frequency of CMV- (H), EBV- (I) and influenza-specific (J) CD8$^+$ T cells (CMV, EBV and influenza respectively: younger CMV$^-$ n=15, n=4 and n=12; younger CMV$^+$ n=16, n=6 and n=6; older CMV$^-$ n=16, n=8 and n=10; older CMV$^+$ n=13, n=7 and n=8). (C-J) Each dot represents an epitope-specific population of an individual donor. Open symbols indicate tetramer$^+$CD8$^+$ T cell populations consisting of 3-8 detected cells, these were excluded from phenotypic analysis. Frequency of tetramer$^+$CD8$^+$ T cells are shifted by 10^{-4} to allow for visibility on logarithmic y axes (i.e. no detected tetramer$^+$ events displayed as 10^{-4}). (A, B) Statistical analysis was performed using a Student's t-test, horizontal lines indicate mean. (C-E, G-J) Statistical analysis was performed using a Wilcoxon rank-sum test including a Bonferroni-Holm's multiple comparison correction, horizontal lines indicate median. (G) Statistical analysis compared severity within a donor group and between donor groups per severity. Significant *p*-values are provided above the graph.

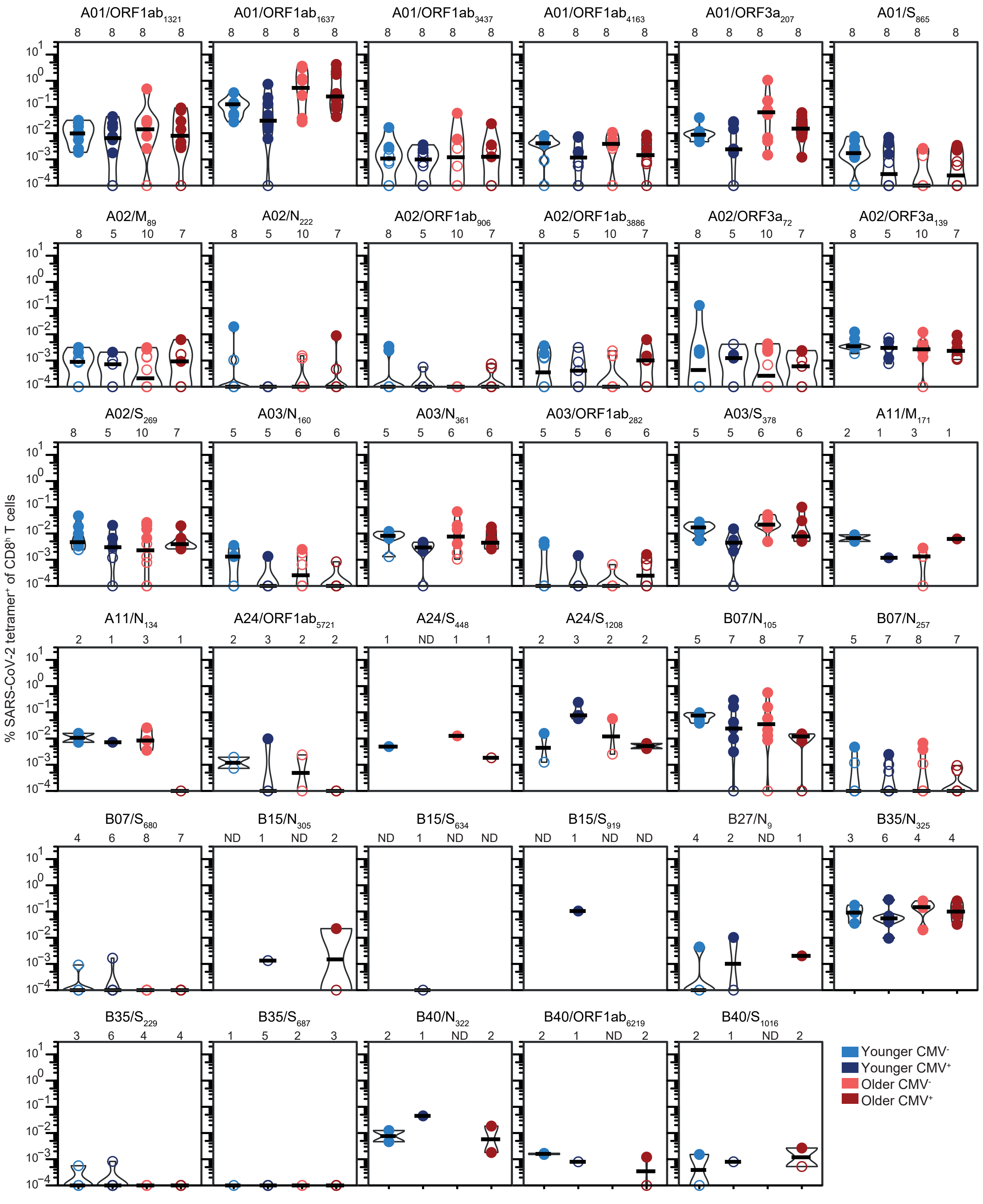

Figure S3. Detected tetramer$^+$CD8$^+$ T cell frequencies per epitope

Dynamics of 35 SARS-CoV-2 epitope-specific CD8$^+$ T cell populations across all donor groups, each dot represents a donor, and the number of donors included is indicated at the top of the graph, not determined (ND) indicates epitopes for which no donors were available. Open symbols represent tetramer$^+$CD8$^+$ T cell populations consisting of 3-8 detected cells, these were excluded from phenotypic analysis. Frequency of tetramer$^+$CD8$^+$ T cells are shifted by 10^{-4} to allow for visibility on logarithmic y axes (i.e. no detected tetramer+ events displayed as 10^{-4}). Statistical analysis was performed using a Wilcoxon rank-sum test including Bonferroni-Holm's multiple comparison correction, horizontal lines indicate median. No significant differences were found.

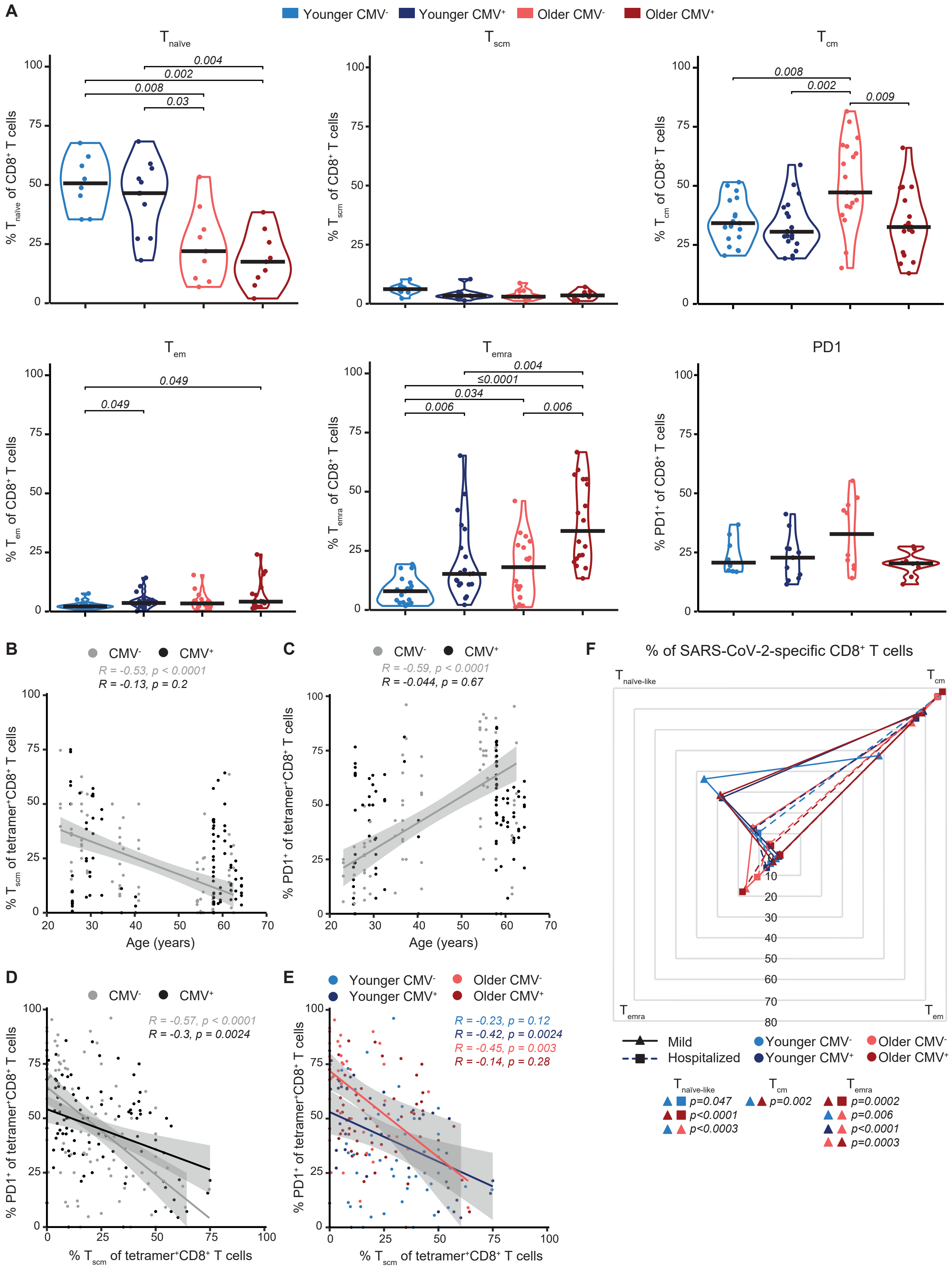

A
Younger CMV-
Younger CMV+
Older CMV-
Older CMV+
T naïve
% T naïve of CD8+ T cells
0.004
0.002
0.008
0.03
T scm
% T scm of CD8+ T cells
T cm
% T cm of CD8+ T cells
0.008
0.002
0.009
T em
% T em of CD8+ T cells
0.049
0.049
T emra
% T emra of CD8+ T cells
0.004
≤0.0001
0.034
0.006
0.006
PD1
% PD1+ of CD8+ T cells
B
CMV-
CMV+
R = -0.53, p < 0.0001
R = -0.13, p = 0.2
% T scm of tetramer+CD8+ T cells
Age (years)
C
CMV-
CMV+
R = -0.59, p < 0.0001
R = -0.044, p = 0.67
% PD1+ of tetramer+CD8+ T cells
Age (years)
D
CMV-
CMV+
R = -0.57, p < 0.0001
R = -0.3, p = 0.0024
% PD1+ of tetramer+CD8+ T cells
% T scm of tetramer+CD8+ T cells
E
Younger CMV-
Younger CMV+
Older CMV-
Older CMV+
R = -0.23, p = 0.12
R = -0.42, p = 0.0024
R = -0.45, p = 0.003
R = -0.14, p = 0.28
% PD1+ of tetramer+CD8+ T cells
% T scm of tetramer+CD8+ T cells
F
% of SARS-CoV-2-specific CD8+ T cells
T naïve-like
T cm
T emra
T em
Mild
Hospitalized
Younger CMV-
Younger CMV+
Older CMV-
Older CMV+
T naïve-like
p=0.047
p<0.0001
p<0.0003
T cm
p=0.002
T emra
p=0.0002
p=0.006
p<0.0001
p=0.0003

Figure S4. Age-associated decrease of $T_{naïve}$ CD8$^+$ T cells frequencies while T_{emra} population increase is age and CMV associated

(A) $T_{naïve}$, T_{scm}, T_{cm}, T_{em}, T_{emra} and PD1 frequencies of the total CD8$^+$ T cell population per donor group (T_{cm}, T_{em}, T_{emra}: younger CMV$^-$ n=19, younger CMV$^+$ n=21, older CMV$^-$ n=19 and older CMV$^+$ n=18 adults; $T_{naïve}$, T_{scm} and PD1: younger CMV$^-$ n=8, younger CMV$^+$ n=11, older CMV$^-$ n=10 and older CMV$^+$ n=9 adults). Statistical analysis was performed using a Wilcoxon rank-sum test along with Bonferroni-Holm's multiple comparison correction. Significant *p*-values are displayed above the graph, horizontal lines indicate median. Spearman's rank-order correlation of the percentage of SARS-CoV-2-specific CD8$^+$ T_{scm} (B) or PD1$^+$ (C) cells with age. (D, E) Spearman's rank-order correlation of the percentage of SARS-CoV-2-specific CD8$^+$PD1$^+$ with SARS-CoV-2-specific CD8$^+$ T_{scm} cells grouped per CMV status (D) or both age and CMV status (E). (B-E) Each dot represents an epitope-specific population of an individual donor (younger CMV$^-$ n=8, younger CMV$^+$ n=10, older CMV$^-$ n=9 and older CMV$^+$ n=9 or pooled per age or CMV status group). Lines indicate significant correlations, the 95% confidence interval is shown in grey. Dots represent SARS-CoV-2 tetramer$^+$CD8$^+$ T cell populations. (F) Radar plot of median phenotype frequencies of the tetramer$^+$CD8$^+$ T cell populations in mild and hospitalized patients per donor group (mild and hospitalized respectively: younger CMV$^-$ n=13 and n=2; younger CMV$^+$ n=15 and n=2; older CMV$^-$ n=11 and n=6 ; older CMV$^+$ n=14 and n=4 adults). Statistical analysis was performed using a Wilcoxon rank-sum test including Bonferroni-Holm's multiple comparison correction to compare severity within a donor group and between donor groups per severity. Significant *p*-values are displayed underneath the radar plots.
