# Supplementary material for: Age and Latent Cytomegalovirus Infection Do Not Affect the Magnitude of De Novo SARS‐CoV‐2‐Specific CD8+ T Cell Responses
Source: Eur J Immunol. 2025 Mar 12;55(3):e202451565. doi: 10.1002/eji.202451565 (PMC11898545; doi:10.1002/eji.202451565)
Supplement: Supplementary file 2 — Supporting Information [file EJI-55-e202451565-s001.docx]

| **Table S1 Convalescent cohort demographics** | | | | | | | | | | | |
| --- | --- | --- | --- | --- | --- | --- | --- | --- | --- | --- | --- |
| Donor ID | Age (years) | Age group | CMV-seroconverted | Sex | COVID-19  severity | Days post symptom onset | HLA-A | HLA-A | HLA-B | HLA-B | Data obtained from van den Dijssel et al. 2022^*^ |
| D04 | 30 | younger | no | M | hospitalized | 29 | **02:01** |  | 18:01 | 38:01 | yes |
| D07 | 27 | younger | no | M | mild | 130 | **02:01** | 26:01 | 38:01 | 49:01 | yes |
| D11 | 31 | younger | no | F | unknown | unknown | 02:01 | **11:01** | **35:01** | 44:03 | yes |
| D16 | 30 | younger | no | M | mild | 143 | **03:01** |  | **07:02** | **35:01** | yes |
| D19 | 24 | younger | no | F | mild | 67 | 32:01 | 68:01 | **40:01** | 44:05 | yes |
| D20 | 32 | younger | no | F | unknown | unknown | **24:02** | 66:01 | **07:02** | **40:01** | yes |
| D23 | 37 | younger | no | F | mild | 27 | **11:01** |  | **27:05** | **35:01** | no |
| D24 | 40 | younger | no | F | mild | 51 | **01:01** | 02:06 | 08:01 | **27:05** | no |
| D31 | 24 | younger | no | F | mild | 36 | **01:01** | 25:01 | 08:01 | 51:01 | yes |
| D33 | 32 | younger | no | F | unknown | unknown | **02:01** | **01:01** | 07:02 | 08:01 | yes |
| D38 | 24 | younger | no | M | mild | 54 | **01:01** | **02:01** | 39:01 | 55:01 | yes |
| D42 | 32 | younger | no | F | unknown | unknown | **02:01** | **03:01** | **07:02** | 40:02 | yes |
| D43 | 35 | younger | no | M | hospitalized | 134 | **03:01** | 29:02 | **07:02** | 44:03 | yes |
| D52 | 23 | younger | no | M | mild | 147 | 68:01 | **01:01** | 08:01 | 51:01 | no |
| D53 | 27 | younger | no | F | mild | 143 | **01:01** | **03:01** | 08:01 | 18:01 | no |
| D54 | 29 | younger | no | M | mild | 141 | **01:01** | 68:01 | **27:05** | 08:01 | no |
| D55 | 25 | younger | no | F | mild | 228 | **02:01** | **01:01** | 08:01 | 27:05 | no |
| D56 | 34 | younger | no | M | mild | 56 | **02:01** | **24:02** | 44:02 | **27:05** | no |
| D57 | 36 | younger | no | F | mild | 36 | **03:01** | **02:01** | 45:01 | **07:02** | no |
| D05 | 22 | younger | yes | F | unknown | unknown | **02:01** | 32:01 | 18:01 | 44:02 | yes |
| D14 | 33 | younger | yes | M | mild | 48 | **03:01** |  | **07:02** | **35:01** | yes |
| D21 | 28 | younger | yes | M | mild | 58 | 02:06 | **24:02** | **27:05** | **40:01** | yes |
| D26 | 28 | younger | yes | F | mild | 36 | 01:01 | **24:02** | 08:01 | 35:03 | yes |
| D29 | 26 | younger | yes | F | mild | 212 | 02:35 | **24:02** | **07:02** | 13:02 | yes |
| D30 | 38 | younger | yes | M | mild | 26 | **01:01** | 25:01 | 08:01 | 18:01 | yes |
| D32 | 22 | younger | yes | F | unknown | unknown | **01:01** | 29:02 | 08:01 | 44:03 | yes |
| D34 | 29 | younger | yes | F | mild | 30 | **03:01** | 68:01 | 08:01 | **35:01** | no |
| D37 | 37 | younger | yes | M | mild | 190 | **01:01** |  | 13:02 | 57:01 | yes |
| D45 | 26 | younger | yes | F | hospitalized | 38 | **03:01** |  | **07:02** |  | yes |
| D46 | 40 | younger | yes | M | hospitalized | 64 | 02:70 | **03:01** | 38:01 | 41:02 | yes |
| D58 | 25 | younger | yes | M | mild | 54 | **01:01** | **02:01** | **15:01** | **07:02** | no |
| D59 | 40 | younger | yes | M | hospitalized | 152 | **01:01** | **02:01** | 08:01 | 51:01 | no |
| D60 | 25 | younger | yes | M | mild | 179 | **01:01** | 03:02 | 08:01 | **07:02** | no |
| D61 | 37 | younger | yes | F | mild | 40 | **01:01** | 33:01 | 14:02 | 08:01 | no |
| D62 | 25 | younger | yes | F | mild | 271 | **01:01** |  | 08:01 |  | no |
| D63 | 27 | younger | yes | M | mild | 73 | **02:01** |  | **27:05** | 40:02 | no |
| D64 | 30 | younger | yes | F | mild | 189 | **02:01** | 32:01 | **07:02** | 35:01 | no |
| D65 | 29 | younger | yes | F | mild | 36 | **03:01** |  | **35:01** | 39:06 | no |
| D66 | 32 | younger | yes | M | mild | 132 | **11:01** |  | **07:02** | **35:01** | no |
| D67 | 30 | younger | yes | F | mild | 42 | 23:17 | 36:01 | **35:01** | 42:01 | no |
| D03 | 53 | older | no | F | mild | 46 | **02:01** |  | 44:02 | 51:01 | yes |
| D09 | 55 | older | no | M | mild | 228 | 03:01 | **11:01** | **35:01** | 56:01 | yes |
| D13 | 51 | older | no | F | hospitalized | 75 | **03:01** | 23:01 | **35:01** | 44:03 | yes |
| D28 | 59 | older | no | M | mild | 35 | **24:02** | 02:01 | 39:06 | 57:01 | yes |
| D35 | 59 | older | no | F | mild | 353 | **01:01** | **02:01** | 14:01 | 18:01 | yes |
| D36 | 66 | older | no | M | mild | 136 | **01:01** | **02:01** | 35:03 | 57:01 | yes |
| D41 | 61 | older | no | M | hospitalized | 182 | **01:01** | **03:01** | 37:01 |  | yes |
| D48 | 64 | older | no | M | mild | unknown | **01:01** | 29:02 | **07:02** | 44:03 | yes |
| D50 | 59 | older | no | M | hospitalized | 152 | **02:01** |  | **07:02** | 40:02 | yes |
| D68 | 54 | older | no | M | hospitalized | 66 | **01:01** | **02:01** | 44:03 | 08:01 | no |
| D69 | 55 | older | no | M | hospitalized | 52 | **02:01** | **01:01** | 08:01 | 57:01 | no |
| D70 | 54 | older | no | M | hospitalized | 70 | **02:01** |  | **07:02** | 38:01 | no |
| D71 | 56 | older | no | M | hospitalized | 75 | **02:01** |  | 57:01 | **07:02** | no |
| D72 | 55 | older | no | M | mild | 47 | **11:01** | 34:01 | **07:02** | 15:21 | no |
| D73 | 55 | older | no | M | mild | 37 | **01:01** | **02:01** | **07:02** | 51:01 | no |
| D74 | 62 | older | no | F | mild | 240 | **02:01** | **03:01** | **07:02** |  | no |
| D75 | 57 | older | no | M | mild | 48 | **03:01** | **01:01** | 08:01 | **07:02** | no |
| D76 | 62 | older | no | M | mild | 238 | **03:01** | **11:01** | **35:01** |  | no |
| D77 | 61 | older | no | F | mild | 72 | **03:01** | **24:02** | 08:01 | **35:01** | no |
| D01 | 60 | older | yes | F | mild | 63 | **02:01** | 30:01 | 13:02 | 18:01 | yes |
| D10 | 51 | older | yes | F | mild | unknown | 01:01 | **11:01** | 08:01 | 44:02 | yes |
| D17 | 55 | older | yes | M | hospitalized | 77 | 02:01 | **03:01** | **07:02** | **35:01** | yes |
| D25 | 57 | older | yes | F | mild | 32 | 01:01 | **24:02** | 08:01 | 35:02 | yes |
| D39 | 61 | older | yes | M | hospitalized | 69 | **02:01** |  | **15:01** | 44:05 | yes |
| D40 | 62 | older | yes | M | mild | 113 | **01:01** | 32:01 | **15:01** | 37:01 | yes |
| D44 | 51 | older | yes | F | hospitalized | 61 | 32:01 | **03:01** | 40:02 |  | yes |
| D47 | 61 | older | yes | F | mild | 187 | **01:01** | 29:02 | **07:02** | 45:01 | yes |
| D49 | 51 | older | yes | M | mild | 165 | 68:01 | **02:01** | 44:02 | **07:02** | yes |
| D78 | 64 | older | yes | F | mild | 67 | **01:01** | **24:02** | **07:02** | 39:06 | no |
| D79 | 61 | older | yes | F | mild | 56 | **01:01** | 68:02 | 53:01 | **35:01** | no |
| D80 | 59 | older | yes | F | mild | 219 | **02:01** | **01:01** | 44:02 | **35:01** | no |
| D81 | 57 | older | yes | M | mild | 41 | **02:01** | **03:01** | 40:01 | **27:05** | no |
| D82 | 63 | older | yes | M | mild | 55 | **02:01** |  | **40:01** | **07:02** | no |
| D83 | 58 | older | yes | F | mild | 54 | **03:01** | **01:01** | 44:02 | **35:01** | no |
| D84 | 58 | older | yes | M | hospitalized | 64 | **03:01** | **01:01** | 08:01 | **07:02** | no |
| D85 | 60 | older | yes | F | mild | 231 | **03:01** | **01:01** | 44:02 | 14:02 | no |
| D86 | 59 | older | yes | M | mild | 54 | 32:01 | **02:01** | 08:01 | **07:02** | no |
| F = female, M = Male  **Bold** = HLA class I allotypes used for tetramer staining  * van den Dijssel 2022 Clinical & Translational Immunology | | | | | | | | | | | |
