# Supplementary material for: Age and Latent Cytomegalovirus Infection Do Not Affect the Magnitude of De Novo SARS‐CoV‐2‐Specific CD8+ T Cell Responses
Source: Eur J Immunol. 2025 Mar 12;55(3):e202451565. doi: 10.1002/eji.202451565 (PMC11898545; doi:10.1002/eji.202451565)
Supplement: Supplementary file 3 — Supporting Information [file EJI-55-e202451565-s003.docx]

| **Table S2 SARS-CoV-2 epitopes** | | | | | | |
| --- | --- | --- | --- | --- | --- | --- |
| Epitope | HLA-I | Sequence | Length | Protein | Amino acid location | Literature |
| A01/ORF1ab_1321_ | A*01:01 | PTDNYITTY | 9 | ORF1ab | 1321-1329 | [1] |
| A01/ORF1ab_1637_ | A*01:01 | TTDPSFLGRY | 10 | ORF1ab | 1637-1646 | [1] |
| A01/ORF1ab_3437_ | A*01:01 | GTDLEGNFY | 9 | ORF1ab | 3437-3445 | [1] |
| A01/ORF1ab_4163_ | A*01:01 | CTDDNALAYY | 10 | ORF1ab | 4163-4172 | [1] |
| A01/ORF3a_207_ | A*01:01 | FTSDYYQLY | 9 | ORF3a | 207-215 | [1] |
| A01/S_865_ | A*01:01 | LTDEMIAQY | 9 | S | 865-873 | [1] |
| A02/M_89_ | A*02:01 | GLMWLSYFI | 9 | M | 89-97 | [1] |
| A02/N_222_ | A*02:01 | LLLDRLNQL | 9 | N | 222-230 | [1] |
| A02/ORF1ab_906_ | A*02:01 | YLFDESGEFKL | 11 | ORF1ab | 906-916 | [1] |
| A02/ORF1ab_3886_ | A*02:01 | KLWAQCVQL | 9 | ORF1ab | 3886-3894 | [1] |
| A02/ORF3a_72_ | A*02:01 | ALSKGVHFV | 9 | ORF3a | 72-80 | [1] |
| A02/ORF3a_139_ | A*02:01 | LLYDANYFL | 9 | ORF3a | 139-147 | [1] |
| A02/S_269_ | A*02:01 | YLQPRTFLL | 9 | S | 269-277 | [1] |
| A03/N_361_ | A*03:01 | KTFPPTEPK | 9 | N | 361-369 | [1] |
| A03/N_160_ | A*03:01 | QLPQGTTLPK | 10 | N | 160-169 | [1] |
| A03/ORF1ab_282_ | A*03:01 | KTIQPRVEK | 9 | ORF1ab | 282-290 | [1] |
| A03/S_378_ | A*03:01 | KCYGVSPTK | 9 | S | 378-386 | [1] |
| A11/M_171_ | A*11:01 | ATSRTLSYYK | 10 | M | 171-180 | [1] |
| A11/N_134_ | A*11:01 | ATEGALNTPK | 10 | N | 134-143 | [1] |
| A24/ORF1ab_5721_ | A*24:02 | VYIGDPAQL | 9 | ORF1ab | 5721-5729 | [1] |
| A24/S_448_ | A*24:02 | NYNYLYRLF | 9 | S | 448-456 | [2]–[5] |
| A24/S_1208_ | A*24:02 | QYIKWPWYI | 9 | S | 1208-1216 | [1] |
| B07/N_105_ | B*07:02 | SPRWYFYYL | 9 | N | 105-113 | [1] |
| B07/N_257_ | B*07:02 | KPRQKRTAT | 9 | N | 257-265 | [1] |
| B07/S_680_ | B*07:02 | SPRRARSVA | 9 | S | 680-688 | [1] |
| B15/N_305_ | B*15:01 | AQFAPSASAF | 10 | N | 305-314 | [1] |
| B15/S_634_ | B*15:01 | RVYSTGSNVF | 10 | S | 634-643 | [6] |
| B15/S_919_ | B*15:01 | NQKLIANQF | 9 | S | 919-927 | [5] |
| B27/N_9_ | B*27:05 | QRNAPRITF | 9 | N | 9-17 | [1] |
| B35/N_325_ | B*35:01 | TPSGTWLTY | 9 | N | 325-333 | [1] |
| B35/S_229_ | B*35:01 | LPIGINITRF | 10 | S | 229-238 | [1] |
| B35/S_687_ | B*35:01 | VASQSIIAY | 9 | S | 687-695 | [6],[7] |
| B40/N_322_ | B*40:01 | MEVTPSGTWL | 10 | N | 322-331 | [1] |
| B40/ORF1ab_6219_ | B*40:01 | IEYPIIGDEL | 10 | ORF1ab | 6219-6228 | [1] |
| B40/S_1016_ | B*40:01 | AEIRASANL | 9 | S | 1016-1024 | [1] |
| S = Spike protein, N = Nucleocapsid protein, M = Membrane protein | | | | | | |

**References**

1. Dijssel J van den, Hagen RR, Jongh R de, Steenhuis M, Rispens T, Geerdes DM, Mok JY, et al. Parallel detection of SARS‐CoV‐2 epitopes reveals dynamic immunodominance profiles of CD8 + T memory cells in convalescent COVID‐19 donors. Clin Transl Immunology. 2022; 11:e1423. DOI: 10.1002/CTI2.1423.

2. Kared H, Redd AD, Bloch EM, Bonny TS, Sumatoh H, Kairi F, Carbajo D, et al. SARS-CoV-2-specific CD8+ T cell responses in convalescent COVID-19 individuals. Journal of Clinical Investigation. 2021; 131:e145476.DOI: 10.1172/JCI145476.

3. Francis JM, Leistritz-Edwards D, Dunn A, Tarr C, Lehman J, Dempsey C, Hamel A, et al. Allelic variation in class I HLA determines CD8+ T cell repertoire shape and cross-reactive memory responses to SARS-CoV-2. Sci. Immunol. 2022; 7:eabk3070.DOI: 10.1126/SCIIMMUNOL.ABK3070.

4. Gangaev A, Ketelaars SLC, Isaeva OI, Patiwael S, Dopler A, Hoefakker K, De Biasi S, et al. Identification and characterization of a SARS-CoV-2 specific CD8+ T cell response with immunodominant features. Nat Commun. 2021; 12:2593.DOI: 10.1038/s41467-021-22811-y.

5. Minervina AA, Pogorelyy M V., Kirk AM, Crawford JC, Allen EK, Chou CH, Mettelman RC, et al. SARS-CoV-2 antigen exposure history shapes phenotypes and specificity of memory CD8+ T cells. Nature Immunology 2022 23:5. 2022; 23:781–790. DOI: 10.1038/s41590-022-01184-4.

6. Tarke A, Sidney J, Kidd CK, Dan JM, Ramirez SI, Yu ED, Mateus J, et al. Comprehensive analysis of T cell immunodominance and immunoprevalence of SARS-CoV-2 epitopes in COVID-19 cases. Cell Rep Med. 2021; 2:100204. DOI: 10.1016/j.xcrm.2021.100204.

7. Saini SK, Hersby DS, Tamhane T, Povlsen HR, Amaya Hernandez SP, Nielsen M, Gang AO, et al. SARS-CoV-2 genome-wide T cell epitope mapping reveals immunodominance and substantial CD8+ T cell activation in COVID-19 patients. Sci Immunol. 2021; 6:eabf7550.DOI: 10.1126/SCIIMMUNOL.ABF7550.
