# Supplementary material for: Age and Latent Cytomegalovirus Infection Do Not Affect the Magnitude of De Novo SARS‐CoV‐2‐Specific CD8+ T Cell Responses
Source: Eur J Immunol. 2025 Mar 12;55(3):e202451565. doi: 10.1002/eji.202451565 (PMC11898545; doi:10.1002/eji.202451565)
Supplement: Supplementary file 4 — Supporting Information [file EJI-55-e202451565-s004.docx]

| **Table S3 Control epitopes** | | | | | | |  |
| --- | --- | --- | --- | --- | --- | --- | --- |
| **Epitope** | **HLA-I** | **Sequence** | **Length** | **Pathogen** | **Protein** | **Amino acid location** | **Literature** |
| A01/pp50_245_ | A*01:01 | VTEHDTLLY | 9 | CMV | pp50 | 245-253 | [1] |
| A02/UL83_495_ | A*02:01 | NLVPMVATV | 9 | CMV | UL83 | 495-503 | [2] |
| A02/M1_58_ | A*02:01 | GILGFVFTL | 9 | Influenza | M1 | 58-66 | [3] |
| B07/pp65_417_ | B*07:02 | TPRVTGGGAM | 10 | CMV | pp65 | 417-426 | [4] |
| B07/EBNA3A_379_ | B*07:02 | RPPIFIRRL | 9 | EBV | EBNA3A | 379-387 | [5] |
| B15/pp65_215_ | B*15:01 | KMQVIGDQY | 9 | CMV | pp65 | 215-223 | [6] |
| B27/NP_383_ | B*27:05 | SRYWAIRTR | 9 | Influenza | NP | 383-391 | [7] |
| B35/pp65_123_ | B*35:01 | IPSINVHHY | 10 | CMV | pp65 | 123-131 | [8] |
| B40/IE1_381_ | B*40:01 | EEAIVAYTL | 9 | CMV | IE1 | 381-389 | [9] |
| B40/LMP2_200_ | B*40:01 | IEDPPFNSL | 9 | EBV | LMP2 | 200-208 | [10] |

**References**

1. Elkington R, Walker S, Crough T, Menzies M, Tellam J, Bharadwaj M, Khanna R. Ex Vivo Profiling of CD8 + -T-Cell Responses to Human Cytomegalovirus Reveals Broad and Multispecific Reactivities in Healthy Virus Carriers . J Virol. 2003; 77:5226–5240. DOI: 10.1128/jvi.77.9.5226-5240.2003.

2. Diamond DJ, York J, Sun JY, Wright CL, Forman SJ. Development of a Candidate HLA A*0201 Restricted Peptide-Based Vaccine Against Human Cytomegalovirus Infection. Blood. 1997; 90:1751–1767.DOI: 10.1182/BLOOD.V90.5.1751.

3. Bednarek MA, Samir ", Sauma,’ Y, Gammon,’ MC, Porter,’ G, Tamhankar S, Williamson’ And AR, et al. The minimum peptide epitope from the influenza virus matrix protein. Extra and Intracellular Loading of HLA-A2. The Journal of Immunology. 1991; 147:4047–4053.

4. Weekes MP, Wills MR, Mynard K, Carmichael AJ, Sissons JGP. The Memory Cytotoxic T-Lymphocyte (CTL) Response to Human Cytomegalovirus Infection Contains Individual Peptide-Specific CTL Clones That Have Undergone Extensive Expansion In Vivo. J Virol. 1999; 73:2099. DOI: 10.1128/JVI.73.3.2099-2108.1999.

5. Hill A, Worth A, Elliott T. Characterization of two Epstein-Barr virus epitopes restricted by HLA-B7*. Eur. J. Immunol. 1995; 25:18–24. DOI: 10.1002/eji.1830250105.

6. Kondo E, Akatsuka Y, Kuzushima K, Tsujimura K, Asakura S, Tajima K, Kagami Y, et al. Identification of novel CTL epitopes of CMV-pp65 presented by a variety of HLA alleles. Blood. 2004; 103:630–638. DOI: 10.1182/BLOOD-2003-03-0824.

7. Carreno BM, Winter CC, Taurog JD, Hansen TH, Biddison WE. Residues in pockets B and F of HLA-B27 are critical in the presentation of an influenza A virus nucleoprotein peptide and influence the stability of peptide – MHC complexes. Int Immunol. 1993; 5:353–360. DOI: 10.1093/INTIMM/5.4.353.

8. Gavin MA, Gilbert MJ, Riddell SR, Greenberg PD, Bevan MJ. Alkali hydrolysis of recombinant proteins allows for the rapid identification of class I MHC-restricted CTL epitopes. The Journal of Immunology. 1993; 151:3971–3980.DOI: 10.4049/jimmunol.151.8.3971.

9. Khan N, Best D, Bruton R, Nayak L, Rickinson AB, Moss PAH. T cell recognition patterns of immunodominant cytomegalovirus antigens in primary and persistent infection. J Immunol. 2007; 178:4455–65. DOI: 10.4049/jimmunol.178.7.4455.

10. Lee SP, Chan ATC, Cheung S-T, Thomas WA, CroomCarter D, Dawson CW, Tsai C-H, et al. CTL Control of EBV in Nasopharyngeal Carcinoma (NPC): EBV-Specific CTL Responses in the Blood and Tumors of NPC Patients and the Antigen-Processing Function of the Tumor Cells. The Journal of Immunology. 2000; 165:573–582. DOI: 10.4049/JIMMUNOL.165.1.573.
